# Knowledge, attitudes, and practices of patients with coronary artery disease and their families regarding coronary artery bypass grafting, multimodal imaging examinations, and postoperative daily management

**DOI:** 10.3389/fcvm.2025.1659150

**Published:** 2026-01-06

**Authors:** Aijia Yu, Gang Liu, Taojun Ren, Jiaying Sun

**Affiliations:** 1 Ultrasound Department, TEDA International Cardiovascular Hospital, Tianjin, China; 2 Cardiac Surgery, TEDA International Cardiovascular Hospital, Tianjin, China

**Keywords:** coronary artery disease, coronary artery bypass grafting, knowledge, attitudes, practice, postoperative care, patient education, multimodal imaging

## Abstract

**Objective:**

To assess the knowledge, attitudes, and practices (KAP) of patients with coronary artery disease (CAD) and their families regarding coronary artery bypass grafting (CABG), multimodal imaging examinations, and postoperative daily management.

**Methods:**

A cross-sectional study was conducted on patients with CAD and their families between January and April 2025, at TEDA International Cardiovascular Hospital, using a self-designed, validated questionnaire.

**Results:**

A total of 512 (96.24%) valid responses were obtained. Among the participants, 338 (66.0%) were CAD patients and 174 (34.0%) were family members. A total of 322 (62.9%) participants were male, and 205 (40.0%) had undergone coronary stenting prior to the current hospitalization. The mean scores for knowledge, attitudes, and practices were 6.26 ± 5.34 (range: 0–26), 28.89 ± 4.10 (range: 8–40), and 32.49 ± 6.23 (range: 10–50), respectively. Multivariable analysis revealed that among patients, higher education, retirement/self-employment, higher income, prior MI hospitalization, and longer CAD duration (>3 years) were associated with better knowledge, which correlated with attitude. Among family members, higher income predicted better knowledge. Better practice was associated with attitude in patients and with attitude, knowledge, female sex, absence of hypertension, and prior MI in family members.

**Conclusion:**

Significant knowledge deficits and suboptimal practices were identified among CAD patients and their families regarding CABG and postoperative management, despite generally positive attitudes. Clinicians and radiologists should prioritize educational programs that address gaps in knowledge and emphasize the interplay between attitudes and practices to enhance postoperative management and long-term outcomes for CAD patients and their families.

## Introduction

Coronary artery disease (CAD) is the leading cause of death in both developed and developing countries (1). Among treatment strategies for CAD, coronary artery bypass grafting (CABG) is a widely recognized and effective approach for coronary artery revascularization (2). Effective self-management after CABG has a significant impact on improving patients' quality of life. Studies have demonstrated that appropriate self-management can lead to marked improvements in quality of life within 3–6 months post-surgery (3).

From a physiological perspective, the management of risk factors such as smoking cessation, weight control, and blood pressure regulation is critical for postoperative recovery. Poor management of these factors can delay improvements in quality of life. From a psychological perspective, maintaining a positive mental state is equally important, as a history of mental illness can adversely affect recovery (4).

Multimodal medical imaging plays a pivotal role in the CABG process, providing critical diagnostic and treatment information for clinicians. Multimodal imaging examinations can assist clinicians in decision-making by evaluating whether coronary artery bypass grafting (CABG) benefits patients with coronary artery disease (5). In the postoperative follow-up phase, multimodal imaging provides complementary insights into patient recovery. Coronary computed tomography angiography (CTA) remains essential for assessing graft patency and reconstructive adequacy (6, 7). Positron emission tomography (PET) and single-photon emission computed tomography (SPECT) continue to evaluate myocardial perfusion (8), while cardiac magnetic resonance imaging (CMR) offers detailed assessments of ventricular function and myocardial viability (9), and transthoracic echocardiography (TTE), a commonly used ultrasound technique, provides real-time evaluation of cardiac structure and function, which is essential in both preoperative assessment and postoperative monitoring (10).

The Knowledge, Attitude, and Practice (KAP) model postulates that individual behavior is shaped by one's knowledge and attitudes. In public health, the study of behavioral practices is often coupled with assessments of knowledge and risk perception, typically conducted through KAP surveys. This model provides a crucial framework for understanding health-related behaviors (11–13). Although the technical and medical aspects of CABG surgery have been extensively studied, limited research focuses on the knowledge, attitudes, and practices (KAP) of patients and their families regarding CABG. Exploring their expectations of surgery, understanding of multimodal imaging examinations, and attitudes toward postoperative management is essential. Recently (14), conducted a cross-sectional study among patients with coronary heart disease and found that most participants had limited understanding of CABG procedures and postoperative management, despite generally positive attitudes toward recovery. Such insights can inform tailored education and support plans, ultimately enhancing the effectiveness of treatment. Illness, particularly CAD, presents challenges not only for patients but also for their families. The onset and progression of the disease profoundly alter the family system and the roles of its members. Consistently, a recent KAP investigation among coronary heart disease patients revealed considerable knowledge gaps regarding antithrombotic therapy, which hindered optimal treatment adherence (15).

This study, therefore, aims to assess the KAP of patients with CAD and their families regarding CABG, multimodal imaging examinations, and postoperative daily management.

## Methods

### Study design and participants

This cross-sectional study was conducted between January and April 2025 at TEDA International Cardiovascular Hospital, focusing on patients with CAD and their family members. Inclusion Criteria: patients with CAD or family mumbers of patients with CAD, determined according to medical history. Eligible patients included those diagnosed with CAD who were scheduled for or had previously undergone coronary artery bypass grafting (CABG) and had completed routine multimodal imaging examinations such as echocardiography, coronary computed tomography angiography, or cardiac magnetic resonance imaging as part of perioperative evaluation. No specific criteria were applied to family members; if a patient met the inclusion criteria, one or more family members could participate upon providing informed consent. Ethical approval was obtained from the Ethics Committee of TEDA International Cardiovascular Hospital, and informed consent was secured from all participants.

### Questionnaire design

The questionnaire was developed based on guidelines (16), refined through feedback from a senior expert, and piloted with a sample of 34 participants, achieving a Cronbach's *α* of 0.900. Administered in Chinese (a version translated into English was attached as Supplementary Data Sheet 1), the final questionnaire covered four key areas. The demographic information section collected data on variables such as age, gender, height, weight, residence, education level, employment status, monthly income, smoking and alcohol consumption habits, exercise patterns, family history of CAD, type of health insurance, and the presence of other medical conditions. Monthly household income per capita was categorized into six levels (<2,000, 2,001–5,000, 5,001–10,000, 10,001–20,000, >20,000, and prefer not to disclose). The cutoff of 2,000 CNY approximately corresponded to the minimum wage standard in China, while 5,000 CNY reflected the personal income tax exemption threshold during the study period, consistent with classifications adopted in recent socioeconomic and public health studies. Age was categorized into three groups (<45, 45–64, and ≥65 years) based on both clinical and epidemiological considerations reflecting the progression of cardiovascular risk across adulthood, corresponding roughly to younger adults (pre-middle age), middle-aged to older middle-aged adults, and elderly individuals. This stratification approach reflects the major life stages relevant to cardiovascular risk profiles and has been adopted in recent cross-sectional studies assessing health behavior and knowledge differences across age groups (17). In addition, subgroup analysis in this study showed distinct gradients in KAP scores across these age categories, supporting the rationality of this grouping. The knowledge dimension assessed participants' understanding of CAD and CABG concepts, perioperative examinations and their functions, as well as the safety and efficacy of CABG. The attitude dimension evaluated perceptions regarding the success rate, effectiveness, potential complications of CABG, the importance of imaging examinations, postoperative rehabilitation, quality of life improvements, and the necessity of follow-up care after surgery. The practice dimension focused on behaviors related to diet, exercise, smoking cessation, medication adherence, weight management, mental health, and regular medical check-ups.

The knowledge dimension included 13 questions scored as follows: “very knowledgeable” (2 points), “have heard of it” (1 point), and “not sure” (0 points), with a total possible score range of 0–26. The attitude dimension consisted of 8 questions rated on a five-point Likert scale from “strongly disagree” (1 point) to “strongly agree” (5 points), yielding a score range of 8–40. Similarly, the practice dimension comprised 10 questions rated on a five-point Likert scale from “never” (1 point) to “always” (5 points), with a total score range of 10–50. Participants who achieved scores above 70% of the maximum in each section were considered to have adequate knowledge, positive attitudes, and proactive practices (18).

### Questionnaire distribution and quality control

Questionnaires were distributed through QR codes in the ultrasound and surgical outpatient and inpatient departments or provided as paper-based versions, using a convenience sampling approach. The internal consistency of the questionnaire, assessed in the main study, was satisfactory for both the overall scale and its subscales. The overall Cronbach's α coefficient was 0.8596, while the coefficients for the knowledge, attitude, and practice sections were 0.9364, 0.7176, and 0.7572, respectively. Content validity was evaluated by a panel of three specialists in cardiovascular medicine and medical education, who assessed the relevance, clarity, and comprehensiveness of each item. These specialists confirmed that all items were correctly formulated, clinically relevant, and aligned with the study objectives. Construct validity was further examined using confirmatory factor analysis (CFA). The Kaiser-Meyer-Olkin (KMO) measure of sampling adequacy for the entire scale was 0.9162, indicating strong suitability for factor analysis. The model demonstrated acceptable fit indices (*χ*^2^/df = 2.61, RMSEA = 0.072, CFI = 0.893, TLI = 0.882), indicating good overall construct validity. Although the RMSEA value slightly exceeded the conventional threshold of 0.06, it remained within the acceptable range (<0.08), suggesting that the scale structure was generally adequate. In addition, the SRMR value (0.087) slightly exceeded the conventional cutoff (<0.08), suggesting minor model residuals; however, this deviation was within an acceptable range and did not substantially affect the overall construct validity.

### Sample size calculation

To determine the required sample size for this cross-sectional study (19), the following formula was used:

$$n=(Z^{2}\times p\times (1-p))/e^{2}$$

Where:

• n is the required sample size,
• Z is the Z-score corresponding to the desired confidence level (1.96 for 95%),
• p is the estimated prevalence (0.5), and
• e is the margin of error (0.05).

The initial calculation yielded a sample size of:

$$n=(1.96^{2}\times 0.5\times (1-0.5))/0.05^{2}=384.$$

To account for a 20% rate of missing or incomplete data, the sample size was adjusted using the formula:

n_adjusted=n/(1−r),

Where r represents the proportion of missing data (0.20). Substituting the values:

n_adjusted=384/(1−0.20)=480.

Thus, the final sample size required was determined to be 480 participants.

### Statistical analysis

Data analysis was performed using SPSS 26.0 (IBM, Armonk, NY, USA) and stata 18.0 (College Station, TX). Continuous variables were presented as means and standard deviations (Mean ± SD), while categorical variables were expressed as frequencies and percentages (*n*, %). Differences in knowledge, attitude, and practice (KAP) scores across demographic subgroups were evaluated using the Mann–Whitney U test for two groups and the Kruskal–Wallis *H*-test for three or more groups, as these variables did not meet normality assumptions. Spearman's correlation analysis was utilized to examine the relationships among knowledge, attitude, and practice scores. Univariate and multivariate logistic regression analyses were performed separately for patients and family members to identify factors associated with each KAP dimension. In the logistic regression analyses, the KAP scores were converted to binary variables according to the median. Structural equation modeling (SEM) was employed to explore the interrelationships among the questionnaire dimensions, with model fit assessed using the root mean square error of approximation (RMSEA), incremental fit index (IFI), Tucker–Lewis index (TLI), and comparative fit index (CFI). A two-sided *P*-value less than 0.05 was considered statistically significant.

## Results

### Demographic information

Initially, 532 cases were collected. After excluding cases with missing consent, abnormal demographic entries, or logical errors, a total of 512 valid responses were included (valid rate: 96.24%). Among all participants, 338 (66.0%) were patients. Table 1 presents the baseline characteristics of the entire study population (*n* = 512). Most participants were male (62.9%) and aged 51–65 years (39.1%).The mean knowledge, attitude, and practice (KAP) scores were 6.26 ± 5.34, 28.89 ± 4.10, and 32.49 ± 6.23, respectively. KAP scores showed significant differences across age, education, employment, and income (all *P* < 0.05). Detailed demographic and clinical characteristics, as well as subgroup comparisons, are presented in Table 1 and Supplementary Table S1.

**Table 1:** Baseline characteristics.

*N* = 512	*N* (%)	Knowledge	*P*	Attitude	*P*	Practice	*P*
		Median [25%, 75%] or mean (SD)		Median [25%, 75%] or mean (SD)		Median [25%, 75%] or mean (SD)	
Total score	512 (100.0)	6.26 (5.34)		28.89 (4.10)		32.49 (6.23)	
Patient/Family member			<0.001		<0.001		<0.001
Patient	338 (66.0)	4.92 (4.11)		28.08 (3.72)		31.22 (5.51)	
Family	174 (34.0)	8.86 (6.40)		30.48 (4.34)		34.95 (6.80)	
Gender			0.634		0.487		0.015
Male	322 (62.9)	6.13 (5.23)		28.85 (3.81)		31.99 (6.22)	
Female	190 (37.1)	6.49 (5.53)		28.97 (4.57)		33.34 (6.18)	
Age			<0.001		<0.001		<0.001
<45 years old	118 (23.0)	9.04 (6.76)		30.33 (4.83)		35.11 (7.24)	
45–64 years old	258 (50.4)	5.94 (4.86)		28.89 (3.74)		32.28 (5.85)	
≥65 years old	136 (26.6)	4.46 (3.63)		27.65 (3.69)		30.60 (5.15)	
Patient's BMI			0.114		0.008		0.117
<23.9	174 (34.0)	6.70 (5.32)		29.51 (4.44)		33.28 (6.05)	
24.0–27.9	223 (43.6)	5.86 (5.37)		28.32 (3.77)		32.09 (6.17)	
≥28.0	115 (22.5)	6.39 (5.31)		29.07 (4.08)		32.07 (6.55)	
Household registration type			0.033		0.862		0.019
Rural	259 (50.6)	5.85 (5.26)		28.79 (4.20)		31.91 (6.30)	
Urban	253 (49.4)	6.69 (5.40)		29.00 (4.00)		33.08 (6.11)	
Education			<0.001		0.002		<0.001
Primary school or below	90 (17.6)	4.29 (4.48)		27.50 (4.44)		29.93 (5.91)	
Middle school	182 (35.5)	5.20 (4.39)		28.68 (3.46)		31.89 (5.67)	
High school/technical school	106 (20.7)	6.56 (5.31)		29.24 (3.67)		33.12 (6.22)	
Associate degree or above	134 (26.2)	8.80 (6.08)		29.84 (4.71)		34.51 (6.48)	
Employment status			<0.001		0.044		0.034
Employed	156 (30.5)	7.61 (6.04)		29.62 (4.45)		33.47 (6.62)	
Retired	129 (25.2)	5.90 (4.82)		28.41 (3.20)		31.89 (4.89)	
Self-employed	53 (10.4)	6.85 (5.72)		29.32 (2.97)		33.02 (6.52)	
Other	175 (34.1)	5.14 (4.62)		28.47 (4.57)		31.89 (6.58)	
Monthly household income per capita, Yuan			<0.001		0.001		<0.001
<2,000	101 (19.7)	4.07 (3.76)		27.80 (4.18)		29.92 (6.18)	
2,001–5,000	231 (45.1)	5.82 (5.01)		28.74 (3.71)		32.37 (5.71)	
5,001–10,000	103 (20.1)	7.78 (5.51)		29.10 (3.69)		33.01 (5.62)	
10,001–20,000	23 (4.5)	7.83 (7.38)		30.04 (5.04)		33.87 (6.82)	
>20,000	13 (2.5)	9.77 (6.11)		33.08 (4.66)		37.92 (6.40)	
Prefer not to disclose	41 (8.0)	8.37 (6.09)		29.95 (5.09)		35.68 (7.47)	
Patient lives alone			0.536		0.314		0.596
Yes	81 (15.8)	6.23 (5.98)		29.28 (3.81)		32.22 (6.56)	
No	431 (84.2)	6.27 (5.22)		28.82 (4.16)		32.54 (6.17)	
Family history of CAD			0.048		0.224		0.453
Yes	132 (25.8)	7.19 (6.06)		29.59 (4.27)		32.58 (6.43)	
No	286 (55.9)	6.19 (5.22)		28.66 (4.10)		32.21 (6.14)	
Uncertain	94 (18.4)	5.18 (4.36)		28.62 (3.77)		33.22 (6.22)	
Type of health insurance (multiple choice)							
Urban employee basic medical insurance	248 (48.4)	6.96 (5.54)		29.27 (3.99)		32.95 (6.14)	
New rural cooperative medical insurance	236 (46.1)	5.53 (5.05)		28.59 (4.19)		32.06 (6.13)	
Urban resident basic medical insurance	23 (4.5)	7.78 (6.84)		29.65 (4.45)		33.26 (6.75)	
Retired cadre medical insurance	4 (0.8)	7.25 (6.70)		27.75 (4.57)		34.75 (6.65)	
Commercial insurance	19 (3.7)	7.26 (5.16)		30.26 (5.87)		33.00 (9.57)	
No insurance	2 (0.4)	2.00 (2.83)		26.50 (3.54)		28.00 (8.49)	
Diabetes			0.12		0.256		0.144
Yes	166 (32.4)	5.67 (4.83)		28.60 (3.60)		31.98 (5.82)	
No	346 (67.6)	6.55 (5.55)		29.03 (4.32)		32.73 (6.41)	
Hypertension			0.052		0.003		<0.001
Yes	266 (52.0)	5.70 (4.81)		28.26 (3.80)		31.28 (6.07)	
No	246 (48.0)	6.87 (5.81)		29.58 (4.31)		33.79 (6.15)	
Obesity			0.256		0.106		0.746
Yes	58 (11.3)	7.86 (7.23)		29.81 (4.24)		32.91 (6.48)	
No	454 (88.7)	6.06 (5.02)		28.78 (4.07)		32.43 (6.20)	
Kidney disease			0.419		0.355		0.815
Yes	12 (2.3)	5.83 (6.55)		29.83 (4.20)		33.17 (5.10)	
No	500 (97.7)	6.27 (5.31)		28.87 (4.10)		32.47 (6.26)	
Liver disease			0.228		0.608		0.608
Yes	11 (2.1)	8.09 (6.20)		29.64 (4.20)		31.91 (6.12)	
No	501 (97.9)	6.22 (5.32)		28.88 (4.10)		32.50 (6.24)	
Stroke			0.397		0.172		0.008
Yes	41 (8.0)	5.24 (4.19)		27.95 (2.72)		29.95 (6.26)	
No	471 (92.0)	6.35 (5.42)		28.97 (4.19)		32.71 (6.18)	
Hospitalized due to heart attack			<0.001		<0.001		<0.001
Yes	178 (34.8)	7.81 (6.24)		30.27 (4.32)		34.28 (7.06)	
No	334 (65.2)	5.44 (4.59)		28.16 (3.79)		31.53 (5.52)	
Duration since CAD diagnosis			0.048		0.435		0.952
With 1 year	258 (50.4)	5.79 (5.00)		28.70 (3.95)		32.55 (6.26)	
1–3 years	75 (14.6)	6.12 (5.52)		28.84 (5.09)		32.21 (6.20)	
Over 3 years	179 (35.0)	7.00 (5.67)		29.20 (3.86)		32.51 (6.23)	
Hospitalized for CAD before this admission (If “No”, skip last 2 questions)			0.245		0.552		0.617
Yes	295 (57.6)	6.57 (5.63)		28.99 (4.03)		32.35 (6.29)	
No	217 (42.4)	5.85 (4.91)		28.76 (4.21)		32.67 (6.15)	
Coronary stent placement before current hospitalization			0.407		0.399		0.53
Yes	205 (40.0)	6.41 (5.56)		28.80 (4.04)		32.12 (6.30)	
No	90 (17.6)	6.91 (5.80)		29.40 (3.99)		32.89 (6.26)	
CABG before current hospitalization			<0.001		<0.001		0.002
Yes	53 (10.4)	9.81 (6.26)		30.94 (4.17)		34.66 (5.83)	
No	242(47.3)	5.86 (5.23)		28.56 (3.87)		31.85 (6.29)	

### Distribution of response to questions of knowledge, attitude, and practice

**Figure 1:**
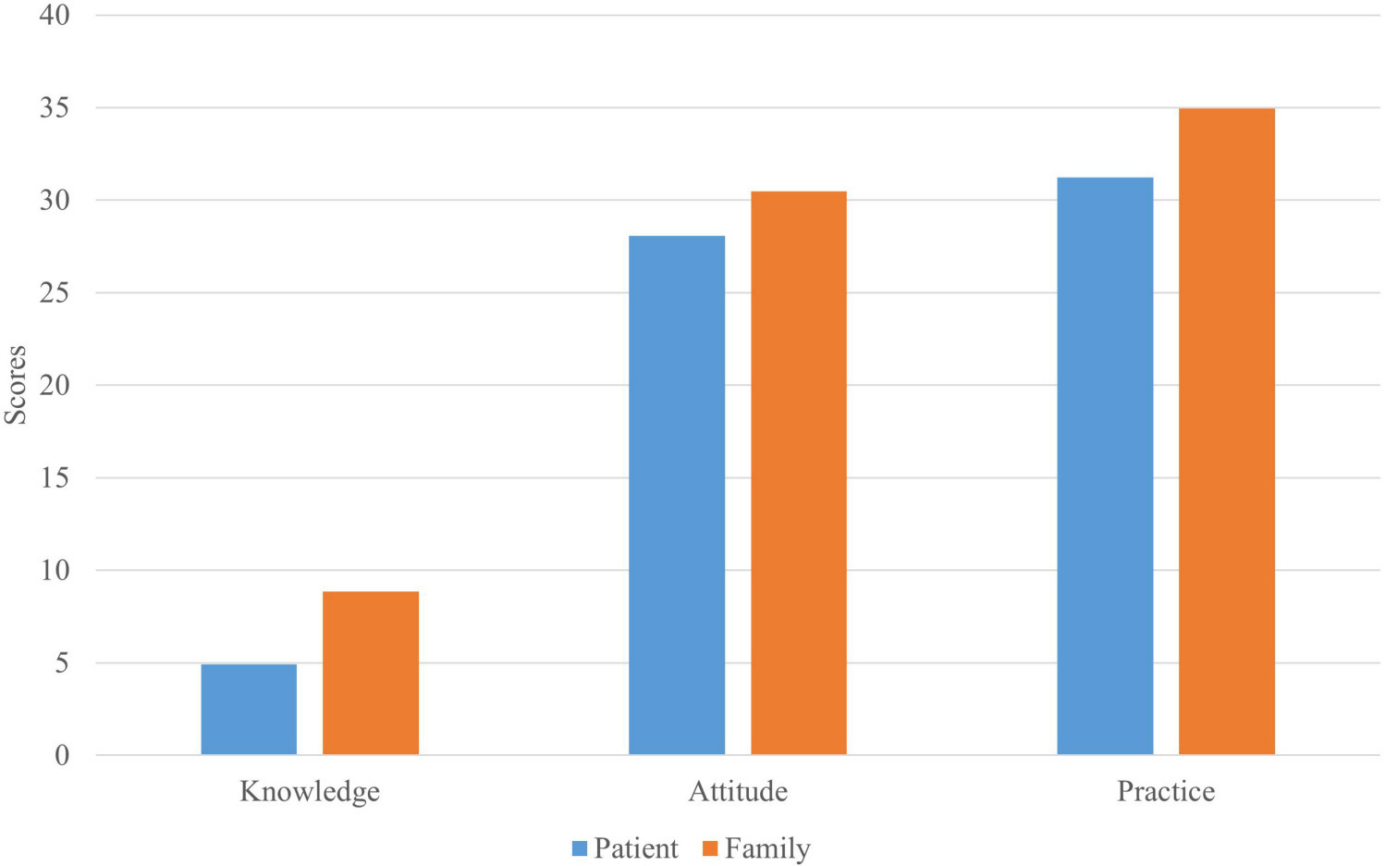
Distribution of total knowledge, attitude, and practice (KAP) scores among patients and family members.

Overall, participants demonstrated limited understanding of CABG-related multimodal imaging and postoperative management. More than half were unfamiliar with cardiac MRI or nuclear imaging, and fewer than 10% reported a good understanding of these modalities. In the knowledge dimension, questions concerning the function and duration of CABG and multimodal imaging had the highest rates of “Unclear” responses. In the attitude dimension, many participants lacked confidence in understanding CABG success rates and potential complications. Regarding practice, nearly one-third rarely sought information about CABG or postoperative management. Detailed distributions of responses for each KAP item are summarized in Supplementary Tables S1–S3. In addition, Figure 1 presents the distributions of total KAP scores with group means, showing that family members scored higher than patients across all domains, knowledge: 8.86 vs. 4.92; attitude: 30.48 vs. 28.08; and practice: 34.95 vs. 31.22.

### Correlations between KAP

Correlation analysis indicated significant positive correlations between knowledge and attitude (*r* = 0.535, *P* < 0.001), as well as practice (*r* = 0.417, *P* < 0.001). Meanwhile, there was also correlation between attitude and practice (*r* = 0.536, *P* < 0.001) (Supplementary Table S4). The distributions of knowledge, attitude, and practice scores showed approximately normal patterns with moderate dispersion across dimensions.

### Univariate and multivariate analysis

In multivariable analyses stratified by respondent type, among patients, higher education (associate degree or above), being retired or self-employed, higher monthly household income (5,001–10,000 CNY), prior hospitalization for heart attack, and longer CAD duration (>3 years) were independently associated with higher knowledge scores (Table 2). Among family members, higher monthly household income (5,001–10,000 CNY) and preferring not to disclose income were associated with higher knowledge (Table 3). Knowledge was independently associated with attitude in both patients and family members (Tables 4, 5). For practice, attitude was independently associated with better practice in both groups (Tables 6, 7); in patients, knowledge showed a borderline association and preferring not to disclose income was significant (Table 6), whereas in family members, knowledge, female sex, absence of hypertension, and prior hospitalization for heart attack were significant (Table 7). Detailed univariate and multivariate regression results are presented in Tables 2–7.

**Table 2:** Univariate and multivariate analysis for knowledge dimension of patients.

Knowledge	Univariate analysis	*P*	Multivariate analysis	*P*
	OR (95% CI)		OR (95% CI)	
Gender				
Male				
Female	0.513 (0.312, 0.831)	0.007	0.892 (0.485, 1.641)	0.713
Age				
<45 years old				
45–64 years old	0.398 (0.163, 0.911)	0.034	0.444 (0.160, 1.232)	0.119
≥65 years old	0.340 (0.136, 0.798)	0.016	0.368 (0.114, 1.183)	0.093
Patient's BMI				
<23.9				
24.0–27.9	1.154 (0.690, 1.938)	0.586		
≥28.0	1.282 (0.709, 2.326)	0.412		
Household registration type				
Rural				
Urban	1.770 (1.150, 2.737)	0.010	0.957 (0.467, 1.961)	0.904
Education				
Primary school or below				
Middle school	2.395 (1.279, 4.651)	0.008	1.309 (0.596, 2.873)	0.503
High school/technical school	3.861 (1.916, 8.042)	<0.001	2.129 (0.874, 5.185)	0.096
Associate degree or above	6.874 (3.273, 15.071)	<0.001	5.346 (1.707, 16.746)	0.004
Employment status				
Employed				
Retired	1.148 (0.628, 2.102)	0.653	3.255 (1.306, 8.109)	0.011
Self-employed	1.287 (0.574, 2.917)	0.541	5.257 (1.660, 16.650)	0.005
Other	0.478 (0.260, 0.874)	0.017	2.303 (0.802, 6.620)	0.121
Monthly household income per capita, Yuan				
<2,000				
2,001–5,000	2.153 (1.219,3.902)	0.010	1.592 (0.801,3.163)	0.184
5,001–10,000	5.252 (2.617, 10.883)	<0.001	2.561 (1.018, 6.442)	0.046
10,001–20,000	3.382 (0.931, 12.821)	0.063	1.433 (0.319, 6.431)	0.639
>20,000	5.636 (1.027, 42.741)	0.055	4.174 (0.596, 29.231)	0.150
Prefer not to disclose	4.026 (1.382, 12.360)	0.012	2.532 (0.743, 8.633)	0.138
Patient lives alone				
Yes				
No	0.968 (0.525, 1.769)	0.917		
Family history of CAD				
Yes				
No	0.703 (0.428, 1.150)	0.161	0.912 (0.520, 1.600)	0.749
Uncertain	0.463 (0.231, 0.908)	0.027	0.618 (0.285, 1.339)	0.222
Diabetes				
Yes				
No	0.963 (0.621, 1.491)	0.866		
Hypertension				
Yes				
No	0.900 (0.576, 1.405)	0.641		
Obesity				
Yes				
No	1.015 (0.518, 1.968)	0.965		
Kidney disease				
Yes				
No	0.489 (0.069, 2.302)	0.396		
Liver disease				
Yes				
No	2.101 (0.507, 10.376)	0.315		
Stroke				
Yes				
No	0.975 (0.471, 1.986)	0.945		
Hospitalized due to heart attack				
Yes				
No	2.434 (1.510, 3.959)	<0.001	2.434 (1.510, 3.959)	<0.001
Duration since CAD diagnosis				
With 1 year				
1–3 years	1.489 (0.802, 2.755)	0.204	1.771 (0.802, 3.910)	0.157
Over 3 years	2.355 (1.443, 3.875)	0.001	2.593 (1.309, 5.139)	0.006
Hospitalized for CAD before this admission (If “No”, skip last 2 questions)				
Yes				
No	1.531 (0.985, 2.395)	0.060	0.966 (0.508, 1.839)	0.917

**Table 3:** Univariate and multivariate analysis for knowledge dimension of the family.

Knowledge	Univariate analysis	*P*	Multivariate analysis	*P*
	OR (95% CI)		OR (95% CI)	
Gender				
Male				
Female	1.273 (0.664, 2.448)	0.467		
Age				
<45 years old				
45–64 years old	0.927 (0.472, 1.832)	0.827		
≥65 years old	0.600 (0.158, 2.507)	0.456		
Patient's BMI				
<23.9				
24.0–27.9	0.663 (0.322, 1.361)	0.263		
≥28.0	0.870 (0.352, 2.261)	0.767		
Household registration type				
Rural				
Urban	1.246 (0.650, 2.400)	0.508		
Education				
Primary school or below				
Middle school	0.628 (0.176, 2.007)	0.446		
High school/technical school	0.682 (0.182, 2.325)	0.550		
Associate degree or above	2.311 (0.636, 7.693)	0.180		
Employment status				
Employed				
Retired	0.546 (0.193, 1.628)	0.260	0.663 (0.213, 2.068)	0.479
Self-employed	1.034 (0.325, 3.976)	0.957	1.271 (0.354, 4.566)	0.713
Other	0.493 (0.233, 1.041)	0.063	0.616 (0.271, 1.396)	0.246
Monthly household income per capita, Yuan				
<2,000				
2,001–5,000	2.800 (0.962, 8.570)	0.062	2.628 (0.880,7.850)	0.083
5,001–10,000	6.735 (1.967, 25.288)	0.003	5.732 (1.562, 21.042)	0.008
10,001–20,000	2.857 (0.636, 14.520)	0.182	2.104 (0.418, 10.587)	0.367
>20,000	/	/	/	/
Prefer not to disclose	4.286 (1.166, 17.366)	0.033	4.330 (1.100, 17.050)	0.036
Patient lives alone				
Yes				
No	0.731 (0.326, 1.704)	0.454		
Family history of CAD				
Yes				
No	0.819 (0.329, 1.907)	0.653		
Uncertain	0.666 (0.236, 1.817)	0.431		
Diabetes				
Yes				
No	0.993 (0.431, 2.444)	0.988		
Hypertension				
Yes				
No	1.033 (0.511, 2.151)	0.930		
Obesity				
Yes				
No	1.121 (0.398, 3.655)	0.837		
Kidney disease				
Yes				
No	0.630 (0.102, 4.893)	0.619		
Liver disease				
Yes				
No	/	/		
Stroke				
Yes				
No	/	/		
Hospitalized due to heart attack				
Yes				
No	1.106 (0.577, 2.136)	0.763		
Duration since CAD diagnosis				
With 1 year				
1–3 years	0.921 (0.326, 2.864)	0.880		
Over 3 years	0.737 (0.366, 1.472)	0.388		
Hospitalized for CAD before this admission (If “No”, skip last 2 questions)				
Yes				
No	0.989 (0.515, 1.895)	0.973		

**Table 4:** Univariate and multivariate analysis for attitude dimension of patients.

Attitude	Univariate analysis	*P*	Multivariate analysis	*P*
	OR (95% CI)		OR (95% CI)	
Knowledge	1.266 (1.165, 1.376)	<0.001	1.266 (1.156,1.386)	<0.001
Gender				
Male				
Female	0.927 (0.577, 1.495)	0.755		
Age				
<45 years old				
45–64 years old	0.923 (0.396, 2.080)	0.847		
≥65 years old	1.080 (0.453, 2.503)	0.858		
Patient's BMI				
<23.9				
24.0–27.9	0.872 (0.518, 1.460)	0.605		
≥28.0	1.160 (0.634, 2.132)	0.630		
Household registration type				
Rural				
Urban	0.919 (0.595, 1.418)	0.702		
Education				
Primary school or below				
Middle school	1.145 (0.646, 2.029)	0.642	0.863 (0.461, 1.614)	0.644
High school/technical school	2.289 (1.157, 4.618)	0.019	1.392 (0.653, 2.968)	0.392
Associate degree or above	1.208 (0.611, 2.401)	0.587	0.692 (0.318, 1.505)	0.353
Employment status				
Employed				
Retired	1.639 (0.886, 3.041)	0.116		
Self-employed	1.730 (0.757, 4.095)	0.201		
Other	1.001 (0.553, 1.809)	0.996		
Monthly household income per capita, Yuan				
<2,000				
2,001–5,000	1.233 (0.721, 2.106)	0.444		
5,001–10,000	1.342 (0.692, 2.630)	0.386		
10,001–20,000	0.991 (0.278, 3.680)	0.989		
>20,000	1.652 (0.305, 12.384)	0.574		
Prefer not to disclose	1.180 (0.413, 3.529)	0.759		
Patient lives alone				
Yes				
No	1.165 (0.634, 2.192)	0.627		
Family history of CAD				
Yes				
No	0.693 (0.414, 1.147)	0.157		
Uncertain	0.720 (0.366, 1.416)	0.339		
Diabetes				
Yes				
No	1.161 (0.746, 1.813)	0.509		
Hypertension				
Yes				
No	1.261 (0.805, 1.976)	0.311		
Obesity				
Yes				
No	1.733 (0.867, 3.661)	0.131		
Kidney disease				
Yes				
No	1.766 (0.375, 12.452)	0.501		
Liver disease				
Yes				
No	1.168 (0.282, 5.773)	0.833		
Stroke				
Yes				
No	0.765 (0.375, 1.572)	0.459		
Hospitalized due to heart attack				
Yes				
No	1.768 (1.084, 2.930)	0.024	1.281 (0.740, 2.217)	0.377
Duration since CAD diagnosis				
With 1 year				
1∼3 years	0.782 (0.424, 1.441)	0.429	0.511 (0.243, 1.072)	0.076
Over 3 years	1.683 (1.019, 2.815)	0.044	0.960 (0.494, 1.866)	0.904
Hospitalized for CAD before this admission (If “No,” skip last 2 questions)				
Yes				
No	1.608 (1.033, 2.508)	0.036	1.581 (0.868, 2.881)	0.135

**Table 5:** Univariate and multivariate analysis for attitude dimension of the family.

Attitude	Univariate analysis	*P*	Multivariate analysis	*P*
	OR (95% CI)		OR (95% CI)	
Knowledge	1.166 (1.083, 1.255)	<0.001	1.164 (1.081,1.252)	<0.001
Gender				
Male				
Female	1.521 (0.777, 3.003)	0.222		
Age				
<45 years old				
45–64 years old	1.018 (0.506, 2.067)	0.960		
≥65 years old	0.537 (0.141, 2.252)	0.367		
Patient's BMI				
<23.9				
24.0–27.9	0.401 (0.185, 0.851)	0.019	0.396 (0.176,0.888)	0.025
≥28.0	0.564 (0.218, 1.508)	0.241	0.543 (0.196,1.501)	0.239
Household registration type				
Rural				
Urban	0.943 (0.481, 1.846)	0.864		
Education				
Primary school or below				
Middle school	1.061 (0.293, 3.484)	0.925		
High school/technical school	1.136 (0.296, 4.046)	0.846		
Associate degree or above	1.497 (0.424, 4.763)	0.506		
Employment status				
Employed				
Retired	0.580 (0.206, 1.727)	0.310		
Self-employed	0.812 (0.268, 2.785)	0.723		
Other	0.989 (0.451, 2.230)	0.979		
Monthly household income per capita, Yuan				
<2,000				
2,001–5,000	0.727 (0.189, 2.323)	0.611		
5,001–10,000	0.811 (0.196, 2.894)	0.756		
10,001–20,000	0.923 (0.163, 5.650)	0.927		
>20,000	/	/		
Prefer not to disclose	0.747 (0.166, 3.038)	0.689		
Patient lives alone				
Yes				
No	2.162 (0.836, 6.727)	0.139		
Family history of CAD				
Yes				
No	0.592 (0.218, 1.442)	0.270		
Uncertain	0.700 (0.225, 2.083)	0.525		
Diabetes				
Yes				
No	1.021 (0.433, 2.616)	0.963		
Hypertension				
Yes				
No	0.737 (0.362, 1.533)	0.405		
Obesity				
Yes				
No	0.958 (0.338, 3.132)	0.938		
Kidney disease				
Yes				
No	/	/		
Liver disease				
Yes				
No	/	/		
Stroke				
Yes				
No	0.477 (0.101, 2.500)	0.345		
Hospitalized due to heart attack				
Yes				
No	1.537 (0.781, 3.083)	0.218		
Duration since CAD diagnosis				
With 1 year				
1–3 years	1.031 (0.349, 3.482)	0.958		
Over 3 years	0.728 (0.354, 1.483)	0.383		
Hospitalized for CAD before this admission (If “No”, skip last 2 questions)				
Yes				
No	1.037 (0.529, 2.029)	0.916		

**Table 6:** Univariate and multivariate analysis for practice dimension of patients.

Practice	Univariate analysis	*P*	Multivariate analysis	*P*
	OR (95% CI)		OR (95% CI)	
Knowledge	1.184 (1.108, 1.265)	<0.001	1.056 (1.001, 1.113)	0.044
Attitude	1.343 (1.227, 1.470)	<0.001	1.265 (1.173, 1.364)	<0.001
Gender				
Male				
Female	0.756 (0.466, 1.215)	0.251		
Age				
<45 years old				
45–64 years old	1.210 (0.537, 2.815)	0.649		
≥65 years old	0.989 (0.428, 2.357)	0.980		
Patient's BMI				
<23.9				
24.0–27.9	0.884 (0.530, 1.476)	0.636		
≥28.0	0.699 (0.383, 1.269)	0.241		
Household registration type				
Rural				
Urban	1.730 (1.122, 2.679)	0.013	1.377 (0.870, 2.180)	0.172
Education				
Primary school or below				
Middle school	1.796 (0.989, 3.334)	0.058	1.033 (0.569, 1.876)	0.915
High school/technical school	2.053 (1.043, 4.103)	0.039	0.990 (0.492, 1.993)	0.977
Associate degree or above	2.976 (1.477, 6.131)	0.003	1.355 (0.629, 2.920)	0.438
Employment status				
Employed				
Retired	1.000 (0.546, 1.832)	0.999		
Self-employed	0.654 (0.280, 1.483)	0.314		
Other	0.792 (0.437, 1.437)	0.442		
Monthly household income per capita, Yuan				
<2,000				
2,001–5,000	1.576 (0.906, 2.790)	0.112	1.488 (0.854, 2.591)	0.161
5,001–10,000	2.454 (1.254, 4.874)	0.009	1.168 (0.570, 2.391)	0.671
10,001–20,000	3.904 (1.083, 16.011)	0.042	1.446 (0.439, 4.763)	0.544
>20,000	2.231 (0.390, 12.768)	0.345	1.385 (0.273, 7.029)	0.694
Prefer not to disclose	5.354 (1.792, 18.295)	0.004	2.948 (1.171, 7.418)	0.022
Patient lives alone				
Yes				
No	0.857 (0.459, 1.571)	0.621		
Family history of CAD				
Yes				
No	0.773 (0.471, 1.269)	0.308		
Uncertain	0.832 (0.426, 1.610)	0.585		
Diabetes				
Yes				
No	0.916 (0.589, 1.421)	0.696		
Hypertension				
Yes				
No	0.737 (0.471, 1.152)	0.181		
Obesity				
Yes				
No	0.765 (0.380, 1.494)	0.440		
Kidney disease				
Yes				
No	0.986 (0.192, 4.540)	0.985		
Liver disease				
Yes				
No	0.431 (0.063, 1.900)	0.307		
Stroke				
Yes				
No	0.515 (0.228, 1.084)	0.092	0.556 (0.266, 1.160)	0.118
Hospitalized due to heart attack				
Yes				
No	1.862 (1.160, 3.002)	0.010	1.429 (0.932, 2.190)	0.102
Duration since CAD diagnosis				
With 1 year				
1∼3 years	1.579 (0.857, 2.921)	0.143		
Over 3 years	1.118 (0.684, 1.822)	0.655		
Hospitalized for CAD before this admission (If “No,” skip last 2 questions)				
Yes				
No	0.925 (0.596,1.438)	0.728		

**Table 7:** Univariate and multivariate analysis for practice dimension of the family.

Practice	Univariate analysis	*P*	Multivariate analysis	*P*
	OR (95% CI)		OR (95% CI)	
Knowledge	1.098 (1.033, 1.168)	<0.001	1.076 (1.022, 1.133)	0.006
Attitude	1.221 (1.109, 1.345)	<0.001	1.241 (1.153, 1.335)	<0.001
Gender				
Male				
Female	3.694 (1.856, 7.648)	<0.001	1.724 (1.136, 2.618)	0.011
Age				
<45 years old				
45–64 years old	0.784 (0.393, 1.565)	0.488		
≥65 years old	0.338 (0.087, 1.315)	0.109		
Patient's BMI				
<23.9				
24.0–27.9	1.092 (0.524, 2.306)	0.815		
≥28.0	0.847 (0.351, 2.130)	0.717		
Household registration type				
Rural				
Urban	1.252 (0.649, 2.432)	0.503		
Education				
Primary school or below				
Middle school	0.377 (0.079, 1.355)	0.165		
High school/technical school	0.577 (0.115, 2.285)	0.458		
Associate degree or above	0.705 (0.150, 2.497)	0.616		
Employment status				
Employed				
Retired	0.731 (0.263, 2.156)	0.554		
Self-employed	1.385 (0.442, 5.276)	0.598		
Other	1.246 (0.578, 2.775)	0.580		
Monthly household income per capita, Yuan				
<2,000				
2,001–5,000	1.579 (0.488, 4.767)	0.425		
5,001–10,000	0.909 (0.266, 2.921)	0.875		
10,001–20,000	1.091 (0.230, 5.499)	0.913		
>20,000	/	/		
Prefer not to disclose	1.636 (0.416, 6.529)	0.477		
Patient lives alone				
Yes				
No	0.816 (0.360, 1.948)	0.633		
Family history of CAD				
Yes				
No	1.360 (0.585, 3.068)	0.464		
Uncertain	1.461 (0.536, 4.057)	0.459		
Diabetes				
Yes				
No	1.132 (0.482, 2.893)	0.783		
Hypertension				
Yes				
No	0.342 (0.170, 0.687)	0.003	0.514 (0.346, 0.764)	0.001
Obesity				
Yes				
No	1.054 (0.373, 3.440)	0.924		
Kidney disease				
Yes				
No	0.595 (0.096, 4.622)	0.576		
Liver disease				
Yes				
No	0.803 (0.075, 17.517)	0.859		
Stroke				
Yes				
No	1.008 (0.209, 7.208)	0.992		
Hospitalized due to heart attack				
Yes				
No	2.005 (1.024, 4.036)	0.046	1.590 (1.034, 2.443)	0.035
Duration since CAD diagnosis				
With 1 year				
1∼3 years	1.046 (0.373, 3.234)	0.935		
Over 3 years	1.075 (0.533, 2.177)	0.839		
Hospitalized for CAD before this admission (If “No,” skip last 2 questions)				
Yes				
No	1.546 (0.800, 3.016)	0.197		

### Interactions between KAP

**Figure 2:**
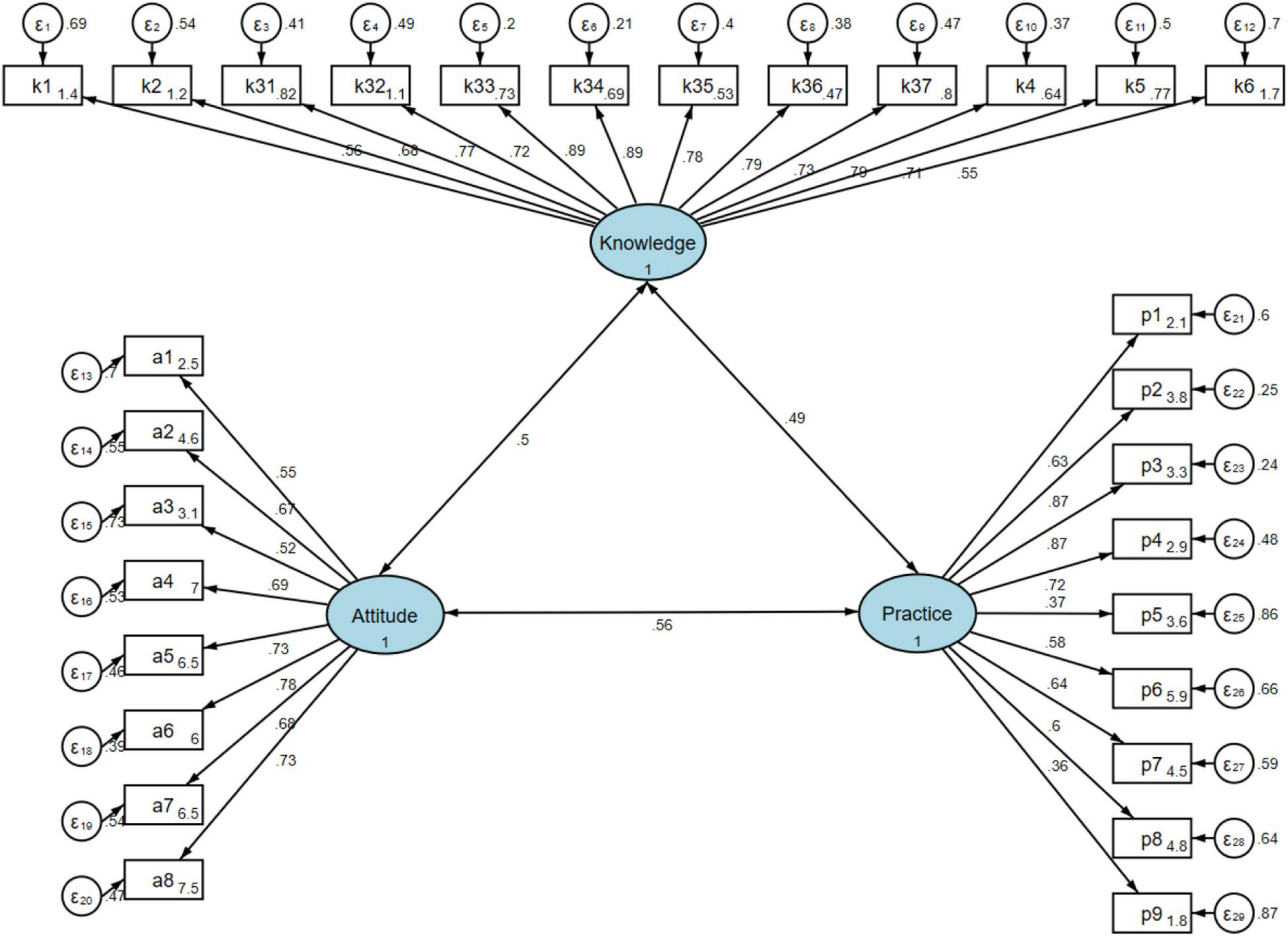
SEM model.

The structural equation model (SEM) demonstrated good model fit (RMSEA = 0.072, SRMR = 0.087, TLI = 0.882, CFI = 0.893). Knowledge directly influenced both attitude (*β* = 0.490, *P* < 0.001) and practice (*β* = 0.337, *P* < 0.001), while attitude also had a direct effect on practice (*β* = 0.427, *P* < 0.001). Moreover, attitude mediated the relationship between knowledge and practice (*β* = 0.209, *P* < 0.001). Detailed fit indices and standardized estimates are provided in Supplementary Tables S6–S8 and Figure 2. Family members showed higher KAP scores than patients (all *P* < 0.001), indicating distinct patterns between the two groups.

## Discussion

### General findings and overview

This study revealed significant knowledge deficits among CAD patients and their families concerning CABG, multimodal imaging, and postoperative management, although their attitudes toward treatment were generally favorable. This study highlights the interconnected dynamics among these dimensions, where knowledge influences both attitudes and practices, as supported by correlation and mediation analyses. Despite its single-center design, this study provides a novel contribution by integrating patients and family members within a unified KAP framework and employing SEM to clarify the pathways linking knowledge, attitudes, and practices in the context of CABG. Consistent with previous studies (20, 21), knowledge deficits often hinder the translation of positive attitudes into sustainable health behaviors, underscoring the need for targeted educational interventions and improved patient support systems.

### Knowledge gaps and barriers

The study revealed significant knowledge gaps regarding multimodal cardiac imaging, which plays a crucial role in CAD diagnosis and CABG evaluation. Echocardiography, despite being widely used clinically for its cost-effectiveness and bedside accessibility, was poorly understood by participants. This finding is clinically relevant, given that echocardiography provides essential information for surgical planning and postoperative monitoring (22). Coronary CTA, while offering detailed information about stenosis characteristics and overall atherosclerosis severity (23), was similarly unfamiliar to many participants. Most notably, over 70% were unaware that cardiac MRI and nuclear imaging could provide critical information about myocardial viability and necrosis—factors crucial for determining CABG benefits (24). The higher awareness of coronary angiography (51%) likely reflects greater emphasis on patient education regarding this gold-standard diagnostic procedure, possibly due to its invasive nature and associated risks (25). These findings have significant implications for clinical practice. The low awareness of multimodal imaging examinations may impact patient compliance with preoperative and postoperative imaging protocols. When patients and their families lack understanding of the purpose and importance of various imaging modalities, they may be less likely to fully engage with the recommended imaging schedules. This is particularly crucial for cardiac MRI and nuclear imaging, as these advanced modalities can provide essential information about myocardial viability that directly influences surgical decision-making (26). Furthermore, patients' limited understanding of echocardiography's role in postoperative monitoring may lead to missed opportunities for early detection of complications.

The influence of socioeconomic factors on knowledge was evident in this study. Higher educational attainment and household income were associated with better knowledge scores, which aligns with previous research indicating that socioeconomic status strongly correlates with health literacy (27, 28). Consistent with previous studies, socioeconomic disparities, such as education level and income, significantly influenced participants' knowledge and attitudes toward CABG and postoperative management, suggesting that interventions should prioritize low-literacy and low-income groups. Lower socioeconomic status, characterized by limited income and education, has been consistently associated with poorer access to reliable health information and restricted opportunities for continuous care. These findings further emphasize that educational disparities must be addressed within broader systemic reforms to achieve sustainable improvements. Integrating structured, culturally tailored education programs into routine clinical workflows could significantly improve knowledge levels. For example, preoperative workshops led by multidisciplinary teams, including surgeons, nurses, radiologist, and dietitians, could ensure that patients and their families receive accurate and actionable information. Additionally, using visual aids and digital tools to convey complex medical concepts may further enhance comprehension, particularly among individuals with limited formal education.

### Attitude and practices

Participants demonstrated generally positive attitudes toward CABG and its outcomes, with many expressing confidence in the effectiveness of the procedure and the potential for improved quality of life. These findings are consistent with studies suggesting that optimism and trust in medical interventions are crucial for promoting adherence to postoperative recommendations (29, 30). However, attitudes varied based on health behaviors, such as smoking, which negatively influenced perceptions of CABG and its associated management. This aligns with broader evidence showing that unhealthy behaviors can undermine confidence in medical treatments (31).

The strong relationship between knowledge and attitudes highlights the need to build foundational understanding to reinforce positive perceptions. Attitudes were also directly linked to better practices, suggesting that interventions targeting attitudinal shifts could have cascading effects on behavior. For instance, involving patients in shared decision-making processes may enhance their sense of agency and confidence in adhering to postoperative care plans. Counseling sessions that address fears and misconceptions about CABG risks, coupled with motivational strategies, could further strengthen positive attitudes. Such initiatives should be personalized to account for individual patient profiles and delivered through trusted healthcare professionals to maximize their impact.

Despite generally positive attitudes, participants exhibited suboptimal practices, particularly in areas such as maintaining a balanced diet, adhering to exercise routines, and attending follow-up appointments. These findings mirror those of other studies, which have identified a disconnect between favorable attitudes and consistent health-promoting behaviors in patients with chronic conditions (32, 33). This gap may be partly attributed to structural barriers such as inadequate follow-up support, limited continuity of care, and insufficient patient monitoring after discharge. Behavioral factors including treatment fatigue, low motivation, and perceived lack of professional guidance may further impede adherence. Such multifactorial barriers highlight the importance of an integrated support system that extends beyond hospital-based education. Barriers to effective practices in this study included logistical challenges, insufficient follow-up support, and a lack of accessible resources, particularly in rural areas. In addition, participants with a history of hypertension were more likely to report lower practice scores. This may be explained by the fact that long-term chronic disease management can lead to treatment fatigue, reduced motivation for sustained behavior change, or lower responsiveness to additional health education interventions (34). These individuals might already be overwhelmed with medication regimens and lifestyle modifications, making it more difficult to adopt or maintain new postoperative self-management practices. These systemic issues highlight the need for a more comprehensive approach to patient care that extends beyond the hospital setting.

Families often demonstrated higher levels of engagement in monitoring dietary habits, exercise, and medication adherence. Healthcare providers should consider integrating family-centered approaches into postoperative care plans, offering guidance and resources to help families actively participate in patient recovery. For example, community health programs could provide follow-up support through regular home visits or virtual check-ins to ensure that patients adhere to prescribed practices. The comparison between patients and their family members also revealed different patterns. Family members tended to be more knowledgeable and positive toward postoperative care, while patients' responses were more affected by their own medical experience and emotional stress during treatment. These findings suggest that educational strategies should address both groups' specific needs rather than treating them as a single population.

### Practical implications and health system strategies

The observed interactions among knowledge, attitudes, and practices highlight the need for an integrated and holistic approach to patient care (Table 8). Knowledge serves as the foundation, influencing attitudes that, in turn, drive practices. However, the disconnect observed between attitudes and practices suggests that addressing systemic and individual-level barriers is crucial for achieving sustainable improvements. These findings are consistent with global trends in chronic disease management, which emphasize the need for integrated care models that address both clinical and psychosocial factors (35, 36).

**Table 8:** Key gaps and barriers (from item-level distributions).

Focus area	Evidence from this study	Brief target
Nuclear cardiac imaging (viability)	“Not clear” **80.1%** (Supplementary Table S1, item 3.6)	Explain purpose and when needed
Cardiac MRI (scar/viability)	“Not clear” **76.0%** (Supplementary Table S1, item 3.5)	Clarify role pre/post CABG
Echo for postoperative follow-up	“Not clear” **64.8%** (Supplementary Table S1, item 3.4)	Discharge checklist with timing/meaning
Basic perioperative echo concepts	“Not clear” **61.9%** (Supplementary Table S1, item 3.3)	One-page “what echo shows”
CABG success rate understanding	“Strongly agree/Agree” **30.4%** (Supplementary Table S2, item 1)	Standard script on outcomes/risks
Active information-seeking	“Always/Often” **20.7%**; “Rarely/Never” **53.7%** (Supplementary Table S3, item 1)	QR hub + where to find info
Regular exercise	“Always/Often” **46.7%** (Supplementary Table S3, item 4)	Simple staged home plan
Awareness benchmark: Coronary angiography	“Very familiar/Heard of it” **59.6%** (Supplementary Table S1, item 3.2)	Use as anchor to explain others

The proportion of specific options was displayed in bold font.

Comparisons with healthcare systems in other regions reveal important insights. For example, studies conducted in high-resource settings often report higher levels of adherence to postoperative practices, likely due to better access to education, resources, and coordinated care (37, 38). These differences highlight the need for context-specific solutions tailored to the unique challenges faced by patients in this study.

To address the knowledge gaps observed, healthcare providers should prioritize the development of comprehensive patient education programs. These initiatives should incorporate interactive, culturally sensitive materials and leverage both in-person and digital delivery methods to enhance accessibility. However, while education and awareness are critical, improving patients’ practices also requires addressing logistical, psychological, and systemic barriers, such as limited follow-up access, treatment fatigue, and insufficient coordination between hospital and community-based care. Attitudes can be further strengthened by fostering trust and confidence through personalized counseling and shared decision-making processes. Interventions should also focus on reducing barriers to practice, such as improving access to follow-up care and integrating family members into the recovery process (39, 40).

System-level reforms and policy interventions are essential for addressing the broader barriers to effective practices and disparities in healthcare access. Policymakers should consider implementing systemic reforms to address disparities in healthcare access and resource allocation. Practical strategies to improve postoperative adherence include implementing structured follow-up protocols, such as scheduled reminder systems and digital tracking tools for lifestyle management. Incorporating mobile health applications can facilitate self-monitoring of diet, exercise, and medication adherence. Furthermore, family-centered educational programs may empower caregivers to actively support patients' recovery, ensuring sustained behavioral change and better long-term outcomes. Subsidizing education programs for patients with lower socioeconomic status and investing in community-based support services could significantly improve adherence to recommended practices. Additionally, enhancing the capacity of primary care systems to provide ongoing support for CAD patients could help bridge gaps in postoperative management. These efforts must be accompanied by rigorous evaluation to ensure their effectiveness and sustainability (41, 42). In summary, this study contributes to the growing body of evidence emphasizing the role of patient and family education in optimizing CABG outcomes. By contextualizing the findings within existing international literature, our results highlight not only the universal challenges of patient adherence and health literacy but also the specific gaps in multimodal imaging awareness in the Chinese healthcare context. Strengthening the discussion and presentation of these findings underscores their practical implications for designing culturally tailored educational and behavioral interventions in cardiovascular care.

## Limitations

This study has several limitations. First, the cross-sectional design prevents the establishment of causal relationships between knowledge, attitudes, and practices. Second, as the study was conducted in a single tertiary hospital, the results may not be fully generalizable to other healthcare settings or populations with different sociodemographic backgrounds. Third, the questionnaire did not include certain psychosocial determinants, such as health literacy, psychological status, social support, and information sources, which may have influenced participants' KAP levels. Fourth, while the questionnaire demonstrated overall acceptable construct validity based on confirmatory factor analysis, the SRMR value (0.087) slightly exceeded the conventional threshold of 0.08, indicating minor model residuals. Although this deviation remained within the acceptable range and did not substantially compromise the scale's validity, future studies may benefit from further refinement of the questionnaire structure to optimize model fit. Fifth, self-reported data might introduce recall and social desirability biases, potentially leading to overestimation of knowledge or positive attitudes. Additionally, the cutoffs used for age and income stratifications were based on practical and policy considerations in China, which may limit cross-context comparability. Furthermore, the questionnaire did not differentiate between imaging examinations with substantially different costs, which may have influenced participants' attitudes and practices toward certain modalities. In addition, some questions in the knowledge section contained technical or specialized medical terms, which may have posed comprehension challenges for respondents with limited health literacy, despite the pilot testing and expert review process. Moreover, the characteristics of families (i.e., relationship and caregiver role) were not fully assessed. Despite these limitations, the large sample size and robust statistical analyses strengthen the reliability of the findings. Future research should employ longitudinal and multicenter designs and consider integrating additional psychosocial and behavioral factors to provide a more comprehensive understanding of KAP determinants in patients undergoing CABG.

## Conclusion

In conclusion, this study identified significant knowledge deficits and suboptimal postoperative practices among CAD patients and their families regarding CABG and multimodal imaging examinations, despite generally positive attitudes. Knowledge directly influenced both attitudes and practices, with attitude serving as a mediator in the knowledge-practice pathway. Among patients, socioeconomic factors including education level, employment status, and income were key determinants of knowledge, while attitude was the primary driver of practice behaviors. Among family members, income predicted knowledge, whereas attitude, knowledge, female sex, and absence of hypertension were associated with better practices. Notably, the majority of participants demonstrated poor understanding of cardiac imaging modalities, particularly cardiac MRI and nuclear imaging, which provide critical information for surgical decision-making and postoperative monitoring. These findings emphasize the need for targeted, multi-level educational interventions that address knowledge gaps, reinforce positive attitudes, and overcome systemic barriers to translate awareness into sustained behavioral change. Nonetheless, these conclusions should be interpreted with caution, as the study was conducted in a single tertiary hospital and the results may not fully generalize to broader or more diverse populations.

## Data Availability

The original contributions presented in the study are included in the article/Supplementary Material, further inquiries can be directed to the corresponding author.

## References

[1] Levine GN Bates ER Bittl JA Brindis RG Fihn SD Fleisher LA 2016 ACC/AHA guideline focused update on duration of dual antiplatelet therapy in patients with coronary artery disease: a report of the American college of cardiology/American heart association task force on clinical practice guidelines. J Am Coll Cardiol. (2016) 68:1082–115. 10.1016/j.jacc.2016.03.513 27036918

[2] Giustino G Mehran R. PCI And CABG surgery in 2014: CABG surgery versus PCI in CAD–surgery strikes again!. Nat Rev Cardiol. (2015) 12:75–7. 10.1038/nrcardio.2014.220 25560379

[3] Grady KL Lee R Subačius H Malaisrie SC McGee EC Jr. Kruse J Improvements in health-related quality of life before and after isolated cardiac operations. Ann Thorac Surg. (2011) 91:777–83. 10.1016/j.athoracsur.2010.11.015 21352997

[4] Schmidt-RioValle J Abu Ejheisheh M Membrive-Jiménez MJ Suleiman-Martos N Albendín-García L Correa-Rodríguez M Quality of life after coronary artery bypass surgery: a systematic review and meta-analysis. Int J Environ Res Public Health. (2020) 17:8439. 10.3390/ijerph17228439 33202650 PMC7697861

[5] Ofoegbu CKP Manganyi RM. Off-pump coronary artery bypass grafting; is it still relevant? Curr Cardiol Rev. (2022) 18:e271021197431. 10.2174/1573403(17666211027141043 34711166 PMC9413736

[6] Collet C Onuma Y Andreini D Sonck J Pompilio G Mushtaq S Coronary computed tomography angiography for heart team decision-making in multivessel coronary artery disease. Eur Heart J. (2018) 39:3689–98. 10.1093/eurheartj/ehy581 30312411 PMC6241466

[7] Kawashima H Onuma Y Andreini D Mushtaq S Morel MA Masuda S Successful coronary artery bypass grafting based solely on non-invasive coronary computed tomography angiography. Cardiovasc Revasc Med. (2022) 40s:187–9. 10.1016/j.carrev.2021.09.003 34556432

[8] Driessen RS Danad I Stuijfzand WJ Raijmakers PG Schumacher SP van Diemen PA Comparison of coronary computed tomography angiography, fractional flow reserve, and perfusion imaging for ischemia diagnosis. J Am Coll Cardiol. (2019) 73:161–73. 10.1016/j.jacc.2018.10.056 30654888

[9] Narula J Chandrashekhar Y Ahmadi A Abbara S Berman DS Blankstein R SCCT 2021 expert consensus document on coronary computed tomographic angiography: a report of the society of cardiovascular computed tomography. J Cardiovasc Comput Tomogr. (2021) 15:192–217. 10.1016/j.jcct.2020.11.001 33303384 PMC8713482

[10] Jolobe OM. Point-of-care transthoracic echocardiography. Clin Med. (2021) 21:e428. 10.7861/clinmed.Let.21.4.5 PMC8313220 35192496

[11] Aerts C Revilla M Duval L Paaijmans K Chandrabose J Cox H Understanding the role of disease knowledge and risk perception in shaping preventive behavior for selected vector-borne diseases in Guyana. PLoS Negl Trop Dis. (2020) 14:e0008149. 10.1371/journal.pntd.0008149 32251455 PMC7170267

[12] Liao L Feng H Jiao J Zhao Y Ning H. Nursing assistants’ knowledge, attitudes and training needs regarding urinary incontinence in nursing homes: a mixed-methods study. BMC Geriatr. (2023) 23:39. 10.1186/s12877-023-03762-z 36683023 PMC9867858

[13] Mumena WA. Maternal knowledge, attitude and practices toward free sugar and the associations with free sugar intake in children. Nutrients. (2021) 13:4403. 10.3390/nu13124403 34959955 PMC8706702

[14] Dong W Yang B Li X Zhang R Chen Q Peng W Coronary heart disease Patients’ knowledge, attitudes, and practices regarding coronary artery bypass grafting. Patient Prefer Adherence. (2025) 19:2861–71. 10.2147/ppa.S548589 40955344 PMC12433663

[15] Du Y Cai X Hong X Chen Y Chen C Gong J Knowledge, attitude, and practice of coronary heart disease patients towards antithrombotic therapy. BMC Public Health. (2025) 25:549. 10.1186/s12889-025-21678-8 39930408 PMC11812212

[16] Lawton JS Tamis-Holland JE Bangalore S Bates ER Beckie TM Bischoff JM 2021 ACC/AHA/SCAI guideline for coronary artery revascularization: executive summary: a report of the American college of cardiology/American heart association joint committee on clinical practice guidelines. J Am Coll Cardiol. (2022) 79:197–215. 10.1016/j.jacc.2021.09.005 34895951

[17] Liu Q Huang YJ Zhao L Wang W Liu S He GP Association between knowledge and risk for cardiovascular disease among older adults: a cross-sectional study in China. Int J Nurs Sci. (2020) 7:184–90. 10.1016/j.ijnss.2020.03.008 32685615 PMC7355188

[18] Lee F Suryohusodo AA. Knowledge, attitude, and practice assessment toward COVID-19 among communities in East Nusa Tenggara, Indonesia: a cross-sectional study. Front Public Health. (2022) 10:957630. 10.3389/fpubh.2022.957630 36388283 PMC9659730

[19] Charan J Biswas T. How to calculate sample size for different study designs in medical research? Indian J Psychol Med. (2013) 35:121–6. 10.4103/0253-7176.116232 24049221 PMC3775042

[20] Liu C Liu S. Knowledge of and attitude toward xenotransplantation among medical students in China: a cross-sectional study. Xenotransplantation. (2021) 28:e12654. 10.1111/xen.12654 33051907

[21] Wu X Li H Li X Yang Y. Knowledge, attitude, and practice of non-emergency surgical patients toward anesthesia. Sci Rep. (2024) 14:17763. 10.1038/s41598-024-68808-7 39085629 PMC11291736

[22] Delgado V van der Kley F Schalij MJ Bax JJ. Optimal imaging for planning and guiding interventions in structural heart disease: a multi-modality imaging approach. Eur Heart J Suppl. (2010) 12:E10–23. 10.1093/eurheartj/suq005

[23] Kadire SR Udelson J Budoff MJ. Imaging in coronary artery disease risk stratification. N Engl J Med. (2021) 385:655–7. 10.1056/NEJMclde2104532 34379929

[24] Chinnaiyan KM Safian RD Gallagher ML George J Dixon SR Bilolikar AN Clinical use of CT-derived fractional flow reserve in the emergency department. Cardiovascular Imaging. (2020) 13:452–61. 10.1016/j.jcmg.2019.05.025 31326487

[25] Steinberg RS Dragan A Mehta PK Toleva O. Coronary microvascular disease in women: epidemiology, mechanisms, evaluation, and treatment. Can J Physiol Pharmacol. (2024) 102:594–606. 10.1139/cjpp-2023-0414 38728748

[26] Aljizeeri A Al-Mallah MH. The role of noninvasive cardiac imaging in the management of diseases of the cardiovascular system. In: Gholamrezanezhad A Assadi M Jadvar H, editors. Radiology-Nuclear Medicine Diagnostic Imaging: A Correlative Approach. Hoboken, NJ: John Wiley & Sons (2023). p. 257–84. 10.1002/9781119603627.ch8

[27] Lopes RT Neves ÉTB Dutra LDC Gomes MC Paiva SM Abreu M Socioeconomic status and family functioning influence oral health literacy among adolescents. Rev Saude Publica. (2020) 54:30. 10.11606/s1518-8787.2020054001842 32215538 PMC7069712

[28] Zanobini P Lorini C Caini S Lastrucci V Masocco M Minardi V Health literacy, socioeconomic status and vaccination uptake: a study on influenza vaccination in a population-based sample. Int J Environ Res Public Health. (2022) 19:6925. 10.3390/ijerph19116925 35682508 PMC9180363

[29] Anger M Valovska T Beloeil H Lirk P Joshi GP Van de Velde M PROSPECT guideline for total hip arthroplasty: a systematic review and procedure-specific postoperative pain management recommendations. Anaesthesia. (2021) 76:1082–97. 10.1111/anae.15498 34015859

[30] Feray S Lubach J Joshi GP Bonnet F Van de Velde M. PROSPECT Guidelines for video-assisted thoracoscopic surgery: a systematic review and procedure-specific postoperative pain management recommendations. Anaesthesia. (2022) 77:311–25. 10.1111/anae.15609 34739134 PMC9297998

[31] Urtecho M Wagner B Wang Z VanderPluym JH Halker Singh RB Noyes J A qualitative evidence synthesis of patient perspectives on migraine treatment features and outcomes. Headache. (2023) 63:185–201. 10.1111/head.14430 36602191

[32] Huang R Grol-Prokopczyk H. Health and health behaviors in China: anomalies in the SES-health gradient? SSM Popul Health. (2022) 17:101069. 10.1016/j.ssmph.2022.101069 35313609 PMC8933530

[33] Schutte AE Srinivasapura Venkateshmurthy N Mohan S Prabhakaran D. Hypertension in low- and middle-income countries. Circ Res. (2021) 128:808–26. 10.1161/circresaha.120.318729 33793340 PMC8091106

[34] Konlan KD Shin J. Determinants of self-care and home-based management of hypertension: an integrative review. Glob Heart. (2023) 18:16. 10.5334/gh.1190 36968303 PMC10038107

[35] Osborne MT Shin LM Mehta NN Pitman RK Fayad ZA Tawakol A. Disentangling the links between psychosocial stress and cardiovascular disease. Circ Cardiovasc Imaging. (2020) 13:e010931. 10.1161/circimaging.120.010931 32791843 PMC7430065

[36] Parigi TL D'Amico F Abreu MT Dignass A Dotan I Magro F Difficult-to-treat inflammatory bowel disease: results from an international consensus meeting. Lancet Gastroenterol Hepatol. (2023) 8:853–9. 10.1016/s2468-1253(23)00154-1 37423233

[37] Faggioni T da Silva Ferreira NC Lopes RM Fidalgo-Neto AA Cotta-de-Almeida V Alves LA. Open educational resources in immunology education. Adv Physiol Educ. (2019) 43:103–9. 10.1152/advan.00116.2018 30835146

[38] Friday VE Hunt C. Open educational resources: equitable and affordable nursing education. Nurs Educ Perspect. (2023) 44:303–5. 10.1097/01.Nep.0000000000001180 37594424

[39] Davidson LJ Cleveland JC Welt FG Anwaruddin S Bonow RO Firstenberg MS A practical approach to left main coronary artery disease: JACC state-of-the-art review. J Am Coll Cardiol. (2022) 80:2119–34. 10.1016/j.jacc.2022.09.034 36423996

[40] Jia S Liu Y Yuan J. Evidence in guidelines for treatment of coronary artery disease. Adv Exp Med Biol. (2020) 1177:37–73. 10.1007/978-981-15-2517-9_2 32246443

[41] Fu Z Liu Q Liang J Weng Z Li W Xu J Association between NMR metabolomic signatures of healthy lifestyle and incident coronary artery disease. Eur J Prev Cardiol. (2023) 30:243–53. 10.1093/eurjpc/zwac252 36317303

[42] Smilowitz NR Toleva O Chieffo A Perera D Berry C. Coronary microvascular disease in contemporary clinical practice. Circ Cardiovasc Interv. (2023) 16:e012568. 10.1161/circinterventions.122.012568 37259860 PMC10330260

