# Supplementary material for: Knowledge, attitudes, and practices of patients with coronary artery disease and their families regarding coronary artery bypass grafting, multimodal imaging examinations, and postoperative daily management
Source: Front Cardiovasc Med. 2026 Jan 6;12:1659150. doi: 10.3389/fcvm.2025.1659150 (PMC12816177; doi:10.3389/fcvm.2025.1659150)
Supplement: Supplementary file 1 [file Table1.docx]

**Supplementary materials**

**Table S1 Distribution of knowledge dimension responses**

| **Knowledge** | Very familiar | Heard of it | Not clear |
| --- | --- | --- | --- |
| 1. Coronary artery bypass grafting (CABG) is a surgery that improves blood flow to the heart, used to treat coronary artery disease (CAD). CABG is also known as heart bypass surgery or coronary artery bypass surgery. | 46(9%) | 326(63.7%) | 140(27.3%) |
| 2. Coronary artery disease refers to the narrowing of coronary arteries, the blood vessels supplying oxygen and nutrients to the heart muscle. This condition is caused by the buildup of plaque, a fatty substance (composed of blood fats, cholesterol, calcium, and other substances) inside the artery walls. When this occurs, the coronary arteries narrow or become blocked, limiting the supply of oxygen-rich blood to the heart muscle. | 39(7.6%) | 282(55.1%) | 191(37.3%) |
| 3. Before CABG, the following examinations are conducted to determine which arteries are narrowed, the degree of narrowing, and any potential heart damage: 3.1Coronary CT is a non-invasive examination of the coronary arteries, providing preliminary information about whether the arteries are narrowed and the extent of narrowing. | 33(6.4%) | 192(37.5%) | 287(56.1%) |
| 3.2 Coronary angiography is performed before CABG to clarify the degree and location of coronary artery narrowing. | 50(9.8%) | 255(49.8%) | 207(40.4%) |
| 3.3Echocardiography provides crucial preoperative information, supporting clinical decisions regarding CABG, including details on heart structure, function, valvular regurgitation, and pericardial effusion. | 31(6.1%) | 164(32%) | 317(61.9%) |
| 3.4Echocardiography is also essential for regular follow-up after CABG, offering information on heart structure, function, valvular regurgitation, and pericardial effusion. | 28(5.5%) | 152(29.7%) | 332(64.8%) |
| 3.5 Cardiac MRI provides information on the presence of fibrotic necrosis in ischemic myocardium. | 20(3.9%) | 103(20.1%) | 389(76%) |
| 3.6 Cardiac nuclear imaging helps determine whether ischemic myocardium still has viability, aiding doctors in evaluating the potential benefits of CABG for the patient. | 19(3.7%) | 83(16.2%) | 410(80.1%) |
| 3.7Many coronary artery disease patients also have cerebral vascular stenosis; a head and neck CT can assess the degree of cerebral vascular narrowing, minimizing the risk of postoperative cerebrovascular events. | 25(4.9%) | 190(37.1%) | 297(58%) |
| 5. The duration of CABG surgery depends on the type of bypass performed, the number of bypassed arteries, graft location, medical history, and any other specific requirements. Generally, CABG takes 3 to 6 hours. | 29(5.7%) | 134(26.2%) | 349(68.2%) |
| 6. While CABG is proven to be safe and effective, like any surgery, it may lead to serious complications, including: blood clots (increasing risks of stroke, heart attack, or lung problems), intraoperative or postoperative bleeding, wound infection and bleeding, anesthesia reactions, irregular heartbeat, pneumonia, breathing issues, fever and pain, kidney failure, memory loss, difficulty concentrating, or clear thinking. | 23(4.5%) | 183(35.7%) | 306(59.8%) |
| 7. CABG is not a cure for coronary artery disease. Postoperative lifestyle changes are crucial for recovery, requiring patients to avoid smoking, take cholesterol-lowering medication, exercise appropriately, maintain a healthy weight, limit animal fats, and consume more vegetables, grains, and fruits. | 49(9.6%) | 358(69.9%) | 105(20.5%) |

**Table S2 Distribution of attitude dimension responses**

| **Attitude** | Strongly agree | Agree | Neutral | Disagree | Strongly disagree |
| --- | --- | --- | --- | --- | --- |
| 1. I have a clear understanding of the success rate of CABG surgery.（P） | 33(6.4%) | 123(24%) | 105(20.5%) | 205(40%) | 46(9%) |
| 2. I believe CABG surgery is an effective way to improve my heart health（P） | 41(8%) | 284(55.5%) | 139(27.1%) | 45(8.8%) | 3(0.6%) |
| 3. I am mentally prepared for the potential risks of complications from CABG surgery.(P) | 24(4.7%) | 179(35%) | 128(25%) | 167(32.6%) | 14(2.7%) |
| 4. I fully understand the significance and importance of preoperative imaging examinations. (P) | 46(9%) | 388(75.8%) | 70(13.7%) | 5(1%) | 3(0.6%) |
| 5. I have full confidence in the recovery period following CABG surgery. (P) | 38(7.4%) | 369(72.1%) | 93(18.2%) | 10(2%) | 2(0.4%) |
| 6. I believe the quality of life will improve after CABG surgery. (P) | 39(7.6%) | 343(67%) | 115(22.5%) | 13(2.5%) | 2(0.4%) |
| 7. I think close follow-up and necessary imaging examinations are still required after CABG surgery. (P) | 47(9.2%) | 384(75%) | 66(12.9%) | 12(2.3%) | 3(0.6%) |
| 8. I believe that family members and healthcare professionals can provide adequate support and confidence for the patient’s postoperative recovery. (P) | 57(11.1%) | 397(77.5%) | 51(10%) | 5(1%) | 2(0.4%) |

**Table S3 Distribution of practice dimension responses**

| **Practice** | Always | Often | Sometimes | Rarely | Never |
| --- | --- | --- | --- | --- | --- |
| 1. I actively seek information about CABG and daily postoperative management. (P) | 34(6.6%) | 72(14.1%) | 131(25.6%) | 153(29.9%) | 122(23.8%) |
| 2. I (monitor the patient to) maintain a balanced diet. (P) | 66(12.9%) | 208(40.6%) | 185(36.1%) | 35(6.8%) | 18(3.5%) |
| 3. I (monitor the patient to) maintain a healthy weight. (P) | 66(12.9%) | 196(38.3%) | 170(33.2%) | 48(9.4%) | 32(6.2%) |
| 4. I (monitor the patient to) keep up with regular exercise. (P) | 65(12.7%) | 174(34%) | 148(28.9%) | 84(16.4%) | 41(8%) |
| 5. I (monitor the patient to) avoid smoking. (P) | 299(58.4%) | 114(22.3%) | 35(6.8%) | 35(6.8%) | 29(5.7%) |
| 6. I (monitor the patient to) take medication as scheduled. (P) | 153(29.9%) | 304(59.4%) | 41(8%) | 10(2%) | 4(0.8%) |
| 7. I (monitor the patient to) have regular follow-ups and necessary imaging examinations at 1 month, 3 months, 6 months, etc., post-surgery. (P) | 116(22.7%) | 280(54.7%) | 73(14.3%) | 38(7.4%) | 5(1%) |
| 8. I (monitor the patient to) maintain a positive mindset. (P) | 112(21.9%) | 275(53.7%) | 95(18.6%) | 27(5.3%) | 3(0.6%) |
| 9.1. If I feel unable to continue, I will actively seek help and encouragement from family or healthcare providers. (Patient response). (P) | 30(5.9%) | 128(25%) | 111(21.7%) | 46(9%) | 23(4.5%) |
| 9.2. If I notice the patient is unable to continue, I will actively help and encourage them. (Family response). (P) | 68(13.3%) | 80(15.6%) | 19(3.7%) | 5(1%) | 2(0.4%) |

**Table S4. Correlation analysis**

| **Spearman** | **Knowledge** | **Attitude** | **Practice** |
| --- | --- | --- | --- |
| **Knowledge** | 1.000 |  |  |
| **Attitude** | 0.535 (P<0.001) | 1.000 |  |
| **Practice** | 0.417 (P<0.001) | 0.536 (P<0.001) | 1.000 |

**Table S5. Univariate and multivariate logistic regression analysis**

| **Cutoff value: median** | N (%) |
| --- | --- |
| **For patients (n=338)** |  |
| Knowledge dimension total score |  |
| Knowledge ≥4 | 186(54.87%) |
| Knowledge ≤3 | 152(45.13%) |
| Attitude dimension total score |  |
| Attitude ≥28 | 199(58.70%) |
| Attitude ≤27 | 139(41.30%) |
| Practice dimension total score |  |
| Practice ≥31 | 179(52.73%) |
| Practice ≤30 | 159(47.27%) |
| **For Families (n=174)** |  |
| Knowledge dimension total score |  |
| Knowledge ≥9 | 87(50.00%) |
| Knowledge ≤8 | 87(50.00%) |
| Attitude dimension total score |  |
| Attitude ≥31 | 92(52.87%) |
| Attitude ≤30 | 82(47.13%) |
| Practice dimension total score |  |
| Practice ≥35 | 92(52.87%) |
| Practice ≤34 | 82(47.13%) |

**Table S6. SEM fit indices**

| **Indicators** | **Reference** | **Results** |
| --- | --- | --- |
| RMSEA | <0.08Good | 0.072 |
| SRMR | <0.08Good | 0.087 |
| TLI | >0.8Good | 0.882 |
| CFI | >0.8Good | 0.893 |

**Table S7. SEM total effect estimates**

| **Indicators** |  | **Estimate** | **P>\|z\|** |
| --- | --- | --- | --- |
| Attitude |  |  |  |
|  | Knowledge | 12.62 | <0.001 |
| Practice |  |  |  |
|  | Knowledge | 7.36 | <0.001 |
|  | Attitude | 15.05 | <0.001 |

**Table S8. Mediation analysis**

| **Model paths** | | Total effects | | Direct Effect | | Indirect effect | |
| --- | --- | --- | --- | --- | --- | --- | --- |
|  |  | β(95%CI) | P | β(95%CI) | P | β(95%CI) | P |
| Attitude |  |  |  |  |  |  |  |
|  | Knowledge | 0.490 (0.414,0.567) | <0.001 | 0.490 (0.414,0.567) | <0.001 |  |  |
| Practice |  |  |  |  |  |  |  |
|  | Knowledge | 0.546 (0.425,0.618) | <0.001 | 0.337 (0.247,0.427) | <0.001 | 0.209 (0.154,0.265) | <0.001 |
|  | Attitude | 0.427 (0.337,0.516) | <0.001 | 0.427 (0.337,0.516) | <0.001 |  |  |
