# Supplementary material for: Knowledge, attitudes, and practices of patients with coronary artery disease and their families regarding coronary artery bypass grafting, multimodal imaging examinations, and postoperative daily management
Source: Front Cardiovasc Med. 2026 Jan 6;12:1659150. doi: 10.3389/fcvm.2025.1659150 (PMC12816177; doi:10.3389/fcvm.2025.1659150)
Supplement: Supplementary file 2 [file Datasheet1.docx]

| Questionnaire ID： | | | | |
| --- | --- | --- | --- | --- |
| Dear Patient/Family Member:  We are researchers from TEDA International Cardiovascular Hospital, and we sincerely invite you to participate in our study. This research aims to understand coronary artery bypass grafting (CABG) knowledge, attitudes, and practices, related imaging exams, and post-operative daily management among coronary heart disease (CHD) patients and their family members. This study intends to provide a scientific basis for developing intervention strategies, which could potentially help more people and improve their health outcomes in the future. Furthermore, this questionnaire may enhance patient adherence to auxiliary examinations through patient and family education on CABG and post-operative care, focusing on imaging aspects, which could positively impact patients' prognosis. Your participation is voluntary. This study has been approved by the Ethics Review Committee. If you agree to participate, please refer to the following instructions.  1. Please complete the questionnaire. There are no right or wrong answers; respond based on your actual situation. You may ask us any questions during the completion process. After finishing, please submit the questionnaire promptly.  2. This study is a simple questionnaire survey and will not affect your physical or psychological health. It involves some personal questions, such as gender and age, but all information will be kept strictly confidential (your information will not be disclosed, so feel free to answer).  3. As a participant, you can inquire about study-related information and progress at any time. If you decide to withdraw, please inform us, and your data will not be included in the research results.  Finally, we sincerely thank you for taking the time out of your busy schedule to support our scientific research!  □I am aware of and consent to the use of the collected data for scientific research.  Signature of Informed Consent：  **Date of Participation:** Year ___ Month ___ Day ___ | | | | |
| Part 1 Basic Information | | | | |
| 1. Are you a CHD patient/family member? | | a. Patient | | b. Family member |
| 2. Your gender： | | a. Male | | b. Female |
| 3. Your age： 。 | | | | |
| 4. Patient’s height: ___ cm; weight: ___ kg； | | | | |
| 5. Your household registration type： | | a. Rural  b. Urban | | |
| 6 Education level： | | a. Primary school or below  b. Middle school  c. High school/technical school  d. Associate degree/bachelor’s degree  e. Master’s degree or above | | |
| 7. Employment status： | | a. Employed  b. Retired  c. Self-employed  f. Other | | |
| 8. Average monthly household income per capita in the past year (including in-kind and rental income): ___ yuan | | a.<2000  b.2001-5000  c.5001-10000  d.10001-20000  e.>20000  f. Prefer not to disclose | | |
| 9. Does the patient live alone? | | a. Yes  b. No | | |
| 10. Do you have a family history of coronary heart disease? | | a. Yes  b. No  c. Uncertain | | |
| 11. Do you have a smoking habit? | | a. Never  b. Previously smoked  c. Currently smoke | | |
| 12. Do you have a drinking habit? | | a. Never  b. Previously drank  c. Currently drink | | |
| 13. Do you have a regular exercise habit? | | a. Yes, type of exercise: ___ (I. Tai Chi; II. Yoga; III. Swimming; IV. Other)  b. No | | |
| 14. Type of medical insurance (multiple selections allowed): | | a. Urban employee basic medical insurance  b. New Rural Cooperative Medical Insurance  c. Urban resident basic medical insurance  d. Retired cadre medical insurance  e. Commercial insurance  f. No insurance | | |
| 15. Do you have any underlying diseases? (multiple selections allowed) | | | a. Diabetes  b. Hypertension  c. Obesity  d. Kidney disease  e. Liver disease  f. Stroke  g. Other | |
| 16. Was the patient hospitalized due to a heart attack? | | | a. Yes  b. No | |
| 17. Duration since CHD diagnosis: | | | a. Within 1 year  b. 1-3 years  c. Over 3 years | |
| 18. Has the patient been hospitalized for CHD before this admission? | | | a. Yes, with regular medication (a. Yes, b. No)  b. No (If “No,” skip questions 19 and 20) | |
| 19. Did the patient undergo coronary stent placement before this hospitalization? | | | a. Yes  b. No | |
| 20. Did the patient undergo coronary artery bypass grafting (CABG) surgery before this hospitalization? | | | a. Yes  b. No | |

| Part 2 Knowledge of Coronary Artery Bypass Grafting (CABG), Related Imaging Examinations, and Postoperative Daily Management | | | |
| --- | --- | --- | --- |
| 1. Coronary artery bypass grafting (CABG) is a surgery that improves blood flow to the heart, used to treat coronary artery disease (CAD). CABG is also known as heart bypass surgery or coronary artery bypass surgery. | a. Very familiar | b. Heard of it | c. Not clear |
| 2. Coronary artery disease refers to the narrowing of coronary arteries, the blood vessels supplying oxygen and nutrients to the heart muscle. This condition is caused by the buildup of plaque, a fatty substance (composed of blood fats, cholesterol, calcium, and other substances) inside the artery walls. When this occurs, the coronary arteries narrow or become blocked, limiting the supply of oxygen-rich blood to the heart muscle. | a. Very familiar | b. Heard of it | c. Not clear |
| 3. Before CABG, the following examinations are conducted to determine which arteries are narrowed, the degree of narrowing, and any potential heart damage: |  |  |  |
| 3.1Coronary CT is a non-invasive examination of the coronary arteries, providing preliminary information about whether the arteries are narrowed and the extent of narrowing. | a. Very familiar | b. Heard of it | c. Not clear |
| 3.2 Coronary angiography is performed before CABG to clarify the degree and location of coronary artery narrowing. | a. Very familiar | b. Heard of it | c. Not clear |
| 3.3Echocardiography provides crucial preoperative information, supporting clinical decisions regarding CABG, including details on heart structure, function, valvular regurgitation, and pericardial effusion. | a. Very familiar | b. Heard of it | c. Not clear |
| 3.4Echocardiography is also essential for regular follow-up after CABG, offering information on heart structure, function, valvular regurgitation, and pericardial effusion. | a. Very familiar | b. Heard of it | c. Not clear |
| 3.5 Cardiac MRI provides information on the presence of fibrotic necrosis in ischemic myocardium. | a. Very familiar | b. Heard of it | c. Not clear |
| 3.6 Cardiac nuclear imaging helps determine whether ischemic myocardium still has viability, aiding doctors in evaluating the potential benefits of CABG for the patient. | a. Very familiar | b. Heard of it | c. Not clear |
| 3.7Many coronary artery disease patients also have cerebral vascular stenosis; a head and neck CT can assess the degree of cerebral vascular narrowing, minimizing the risk of postoperative cerebrovascular events. | a. Very familiar | b. Heard of it | c. Not clear |
| 5. The duration of CABG surgery depends on the type of bypass performed, the number of bypassed arteries, graft location, medical history, and any other specific requirements. Generally, CABG takes 3 to 6 hours. | a. Very familiar | b. Heard of it | c. Not clear |
| 6. While CABG is proven to be safe and effective, like any surgery, it may lead to serious complications, including: blood clots (increasing risks of stroke, heart attack, or lung problems), intraoperative or postoperative bleeding, wound infection and bleeding, anesthesia reactions, irregular heartbeat, pneumonia, breathing issues, fever and pain, kidney failure, memory loss, difficulty concentrating, or clear thinking. | a. Very familiar | b. Heard of it | c. Not clear |
| 7. CABG is not a cure for coronary artery disease. Postoperative lifestyle changes are crucial for recovery, requiring patients to avoid smoking, take cholesterol-lowering medication, exercise appropriately, maintain a healthy weight, limit animal fats, and consume more vegetables, grains, and fruits. | a. Very familiar | b. Heard of it | c. Not clear |

| Part 3 Attitudes Toward Coronary Artery Bypass Grafting (CABG), Related Imaging Examinations, and Postoperative Daily Management | | | | | |
| --- | --- | --- | --- | --- | --- |
| 1. I have a clear understanding of the success rate of CABG surgery.（P） | a. Strongly agree | b. Agree | c. Neutral | d. Disagree | e. Strongly disagree |
| 2. I believe CABG surgery is an effective way to improve my heart health（P） | a. Strongly agree | b. Agree | c. Neutral | d. Disagree | e. Strongly disagree |
| 3. I am mentally prepared for the potential risks of complications from CABG surgery.(P) | a. Strongly agree | b. Agree | c. Neutral | d. Disagree | e. Strongly disagree |
| 4. I fully understand the significance and importance of preoperative imaging examinations. (P) | a. Strongly agree | b. Agree | c. Neutral | d. Disagree | e. Strongly disagree |
| 5. I have full confidence in the recovery period following CABG surgery. (P) | a. Strongly agree | b. Agree | c. Neutral | d. Disagree | e. Strongly disagree |
| 6. I believe the quality of life will improve after CABG surgery. (P) | a. Strongly agree | b. Agree | c. Neutral | d. Disagree | e. Strongly disagree |
| 7. I think close follow-up and necessary imaging examinations are still required after CABG surgery. (P) | a. Strongly agree | b. Agree | c. Neutral | d. Disagree | e. Strongly disagree |
| 8. I believe that family members and healthcare professionals can provide adequate support and confidence for the patient’s postoperative recovery. (P) | a. Strongly agree | b. Agree | c. Neutral | d. Disagree | e. Strongly disagree |

| Part 4 Practices Regarding Coronary Artery Bypass Grafting (CABG), Related Imaging Examinations, and Postoperative Daily Management | | | | | |
| --- | --- | --- | --- | --- | --- |
| 1. I actively seek information about CABG and daily postoperative management. (P) | a. Always | b. Often | c. Sometimes | d. Rarely | e. Never |
| 2. I (monitor the patient to) maintain a balanced diet. (P) | a. Always | b. Often | c. Sometimes | d. Rarely | e. Never |
| 3. I (monitor the patient to) maintain a healthy weight. (P) | a. Always | b. Often | c. Sometimes | d. Rarely | e. Never |
| 4. I (monitor the patient to) keep up with regular exercise. (P) | a. Always | b. Often | c. Sometimes | d. Rarely | e. Never |
| 5. I (monitor the patient to) avoid smoking. (P) | a. Always | b. Often | c. Sometimes | d. Rarely | e. Never |
| 6. I (monitor the patient to) take medication as scheduled. (P) | a. Always | b. Often | c. Sometimes | d. Rarely | e. Never |
| 7. I (monitor the patient to) have regular follow-ups and necessary imaging examinations at 1 month, 3 months, 6 months, etc., post-surgery. (P) | a. Always | b. Often | c. Sometimes | d. Rarely | e. Never |
| 8. I (monitor the patient to) maintain a positive mindset. (P) | a. Always | b. Often | c. Sometimes | d. Rarely | e. Never |
| 9.1. If I feel unable to continue, I will actively seek help and encouragement from family or healthcare providers. (Patient response) (P) | a. Always | b. Often | c. Sometimes | d. Rarely | e. Never |
| 9.2. If I notice the patient is unable to continue, I will actively help and encourage them. (Family response) (P) | a. Always | b. Often | c. Sometimes | d. Rarely | e. Never |

| **Thank you again for filling out our questionnaire, the information you provided will be valuable to us in the future!**  **Thank you for filling out our questionnaire！**  If you have any comments and suggestions on this survey, we would be honored to hear your voice  Opinions and Suggestions： （optional）  In order to this questionnaire research can actually play a role in promoting the smooth development of the future return visit, if you are willing to leave your contact information, we would be grateful!  Your phone number： （optional） |
| --- |
